# Supplementary material for: Endogenous clock-mediated regulation of intracellular oxygen dynamics is essential for diazotrophic growth of unicellular cyanobacteria
Source: Nat Commun. 2024 May 2;15:3712. doi: 10.1038/s41467-024-48039-0 (PMC11065991; doi:10.1038/s41467-024-48039-0)
Supplement: Supplementary file 1 — Supplementary Information [file 41467_2024_48039_MOESM1_ESM.pdf]

Supplementary Figure 1

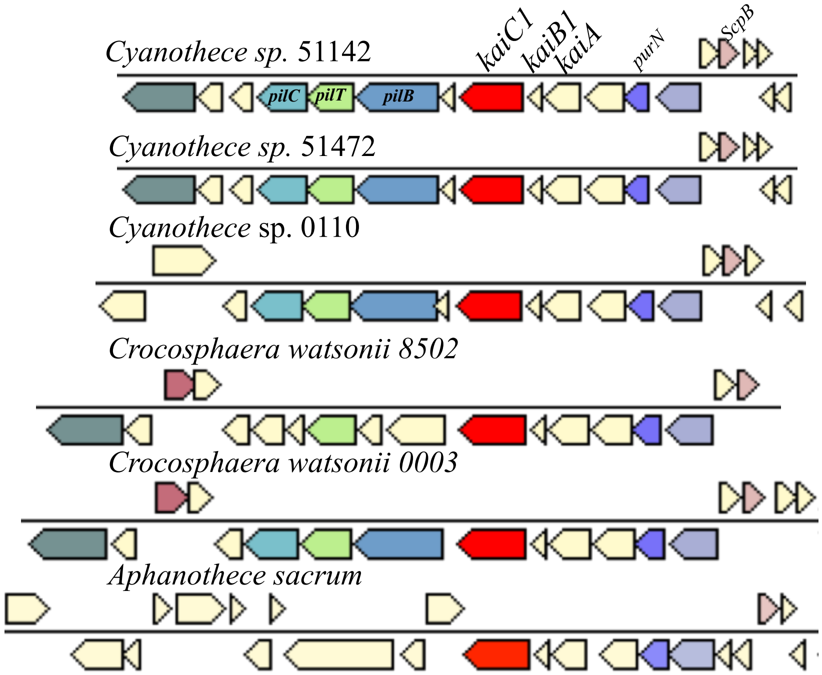

Fig. S1. Alignment of the *Cyanothece* 51142 *kaiABC* gene cluster and its neighborhood. The cluster and its neighborhood are conserved among unicellular diazotrophic cyanobacteria. The alignment was generated with the help of the gene neighborhood tool available at the Integrated Microbial **Genomes** (**IMG**) system (**img**.jgi.doe.gov).

Supplementary Figure 2

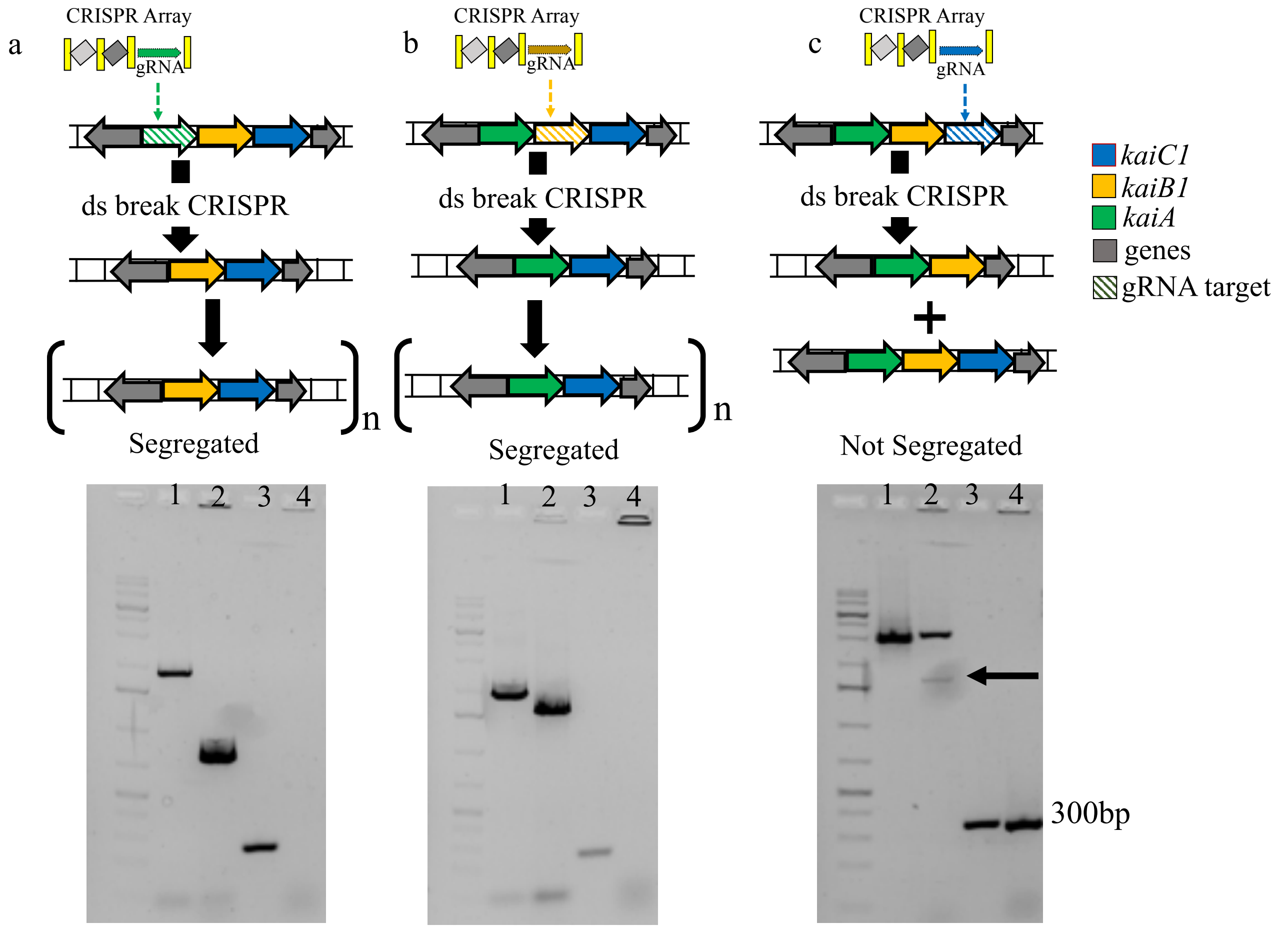

Lane1, 2: WT and clock mutants probed with primers upstream and downstream of the respective genes
Lane3, 4: WT and clock mutants probed with internal gene specific primers

Fig. S2. Generation of clock mutants in *Cyanothece* 51142 using CRISPR-Cas12. Three gRNAs were designed to completely delete individual *kai* genes, *kaiA*, *kaiB* and *kaiC*, leaving the upstream and downstream regions intact. Antibiotic resistant colonies obtained after conjugation were screened by PCR to confirm gene deletion and determine segregation. While Δ*kaiA* (a) and Δ*kaiB* (b) showed complete segregation, Δ*kaiC* (c) retained a WT copy of the gene along with a smaller mutant band (arrow) even after multiple rounds of patching on antibiotic plates.

Supplementary Figure 3

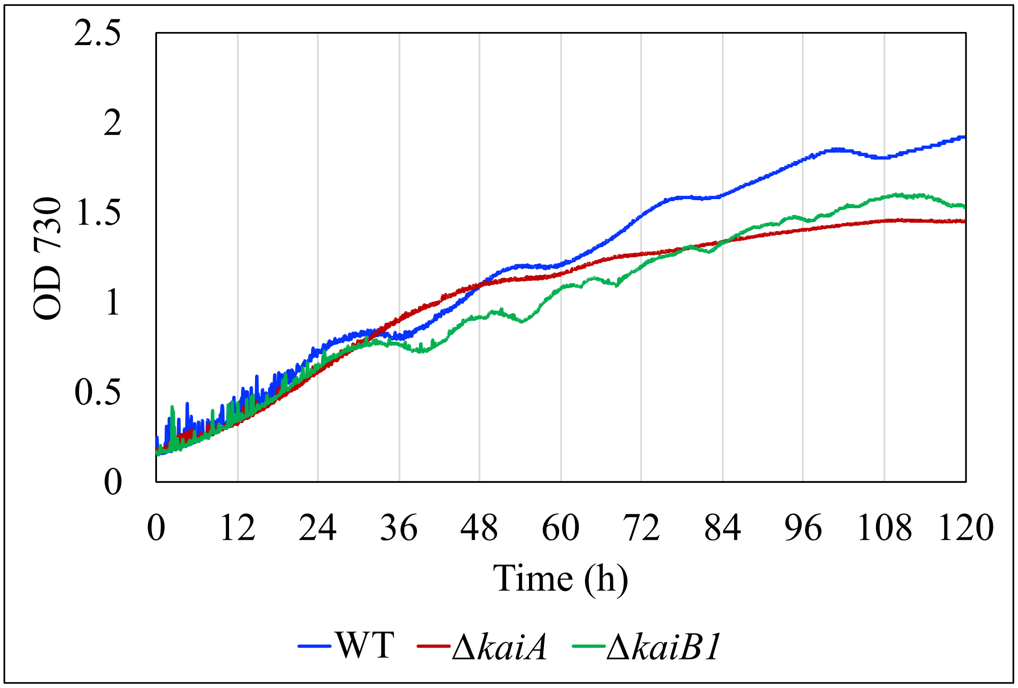

Fig. S3. Deletion of clock genes in *Cyanothece* 51142 leads to distinct growth phenotypes. The WT exhibits prominent endogenous rhythms of ~ 24h (16:8 h phases) under CL and nitrogen fixing conditions. The rhythm is altered in the Δ*kaiB* mutant and is severely dampened /lost in the Δ*kaiA* mutant. Representative curves from 3 independent runs are presented.

Supplementary Figure 4

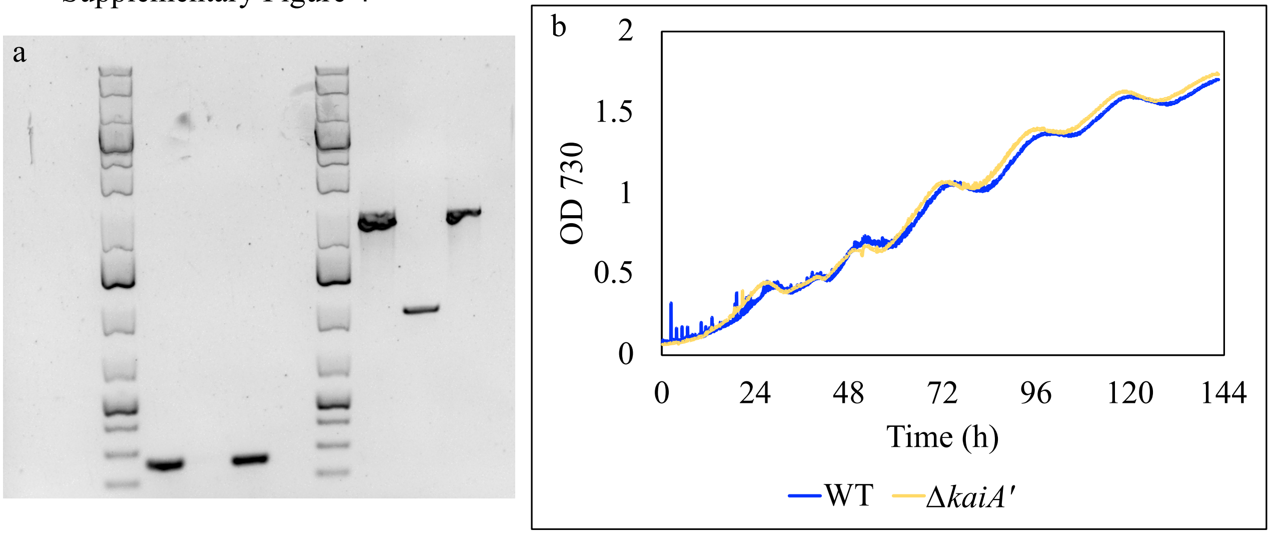

Fig. S4. Complementation of the Δ*kaiA* mutant of *Cyanothece* 51142. (a) PCR confirmation of the complementation. 1-3 - Gene specific primers were used to amplify a 300 bp region within the gene.1– WT, 2 - Δ*kaiA*, 3 - Δ*kaiA*'. 4-6 – Primers specific to regions upstream and downstream of the *kaiA* gene were used. 4 – WT, 5 - Δ*kaiA*, 6 - Δ*kaiA*'. (b) Growth comparison of WT and Δ*kaiA'* complementation strain (Growth curve represents 3 independent runs of the WT and the mutant).

Supplementary Figure 5

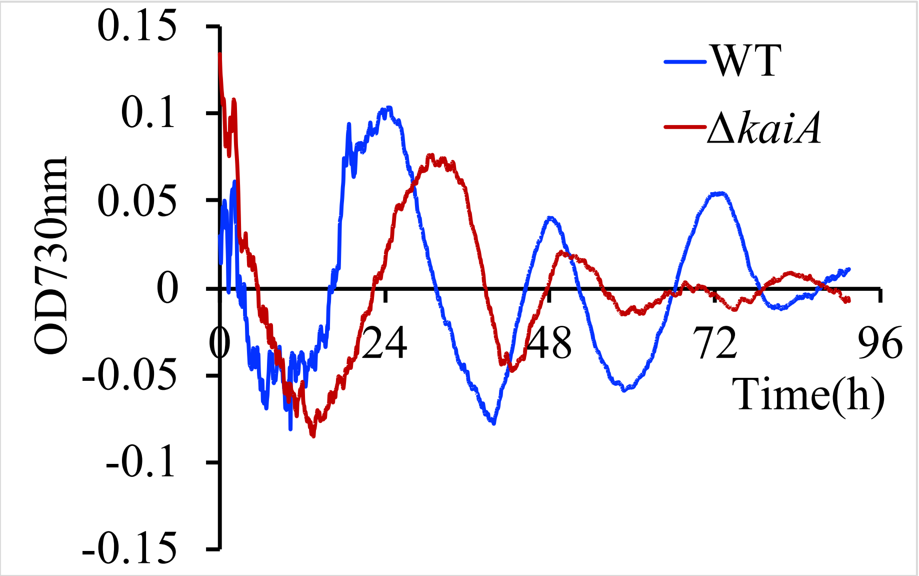

Fig. S5. Detrended growth (measured using OD 730) of the WT and Δ*kaiA* mutant. Detrending was performed using exponential curve fitting on growth curves of multiple WT and Δ*kaiA* samples. Graph shows the mean values for the detrended curves for the two groups.

Supplementary Figure 6

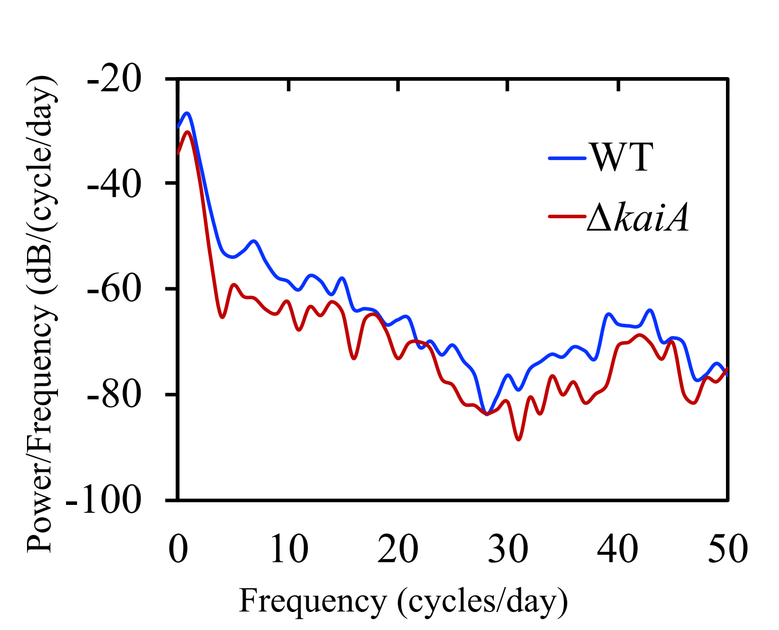

Fig. S6. Quantitative comparison of the periodicity observed in the growth data between the WT and Δ*kaiA* mutant. Power spectrum density (PSD) analysis was performed on the detrended growth data to reveal the PSD at different frequencies.

Supplementary Figure 7

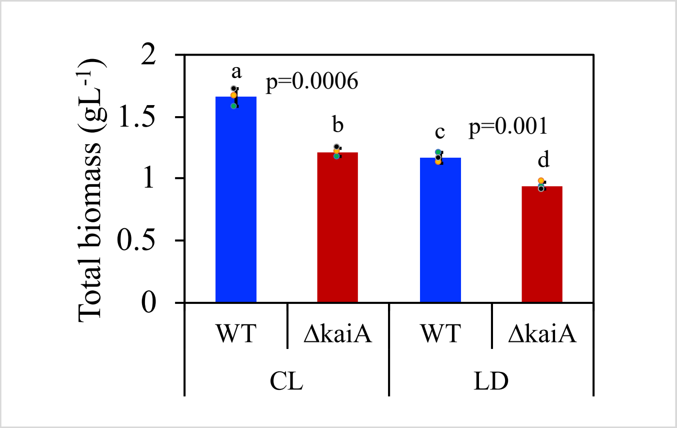

Fig. S7. Comparison of total biomass accumulation in the WT and the Δ*kaiA* mutant under CL and 12:12 LD growth conditions. Samples were collected in triplicate at the end of the growth experiment for final biomass determination as described in the methods section. Error bars correspond to standard deviations from 3 biological replicates (*n*=3 samples).

Supplementary Figure 8

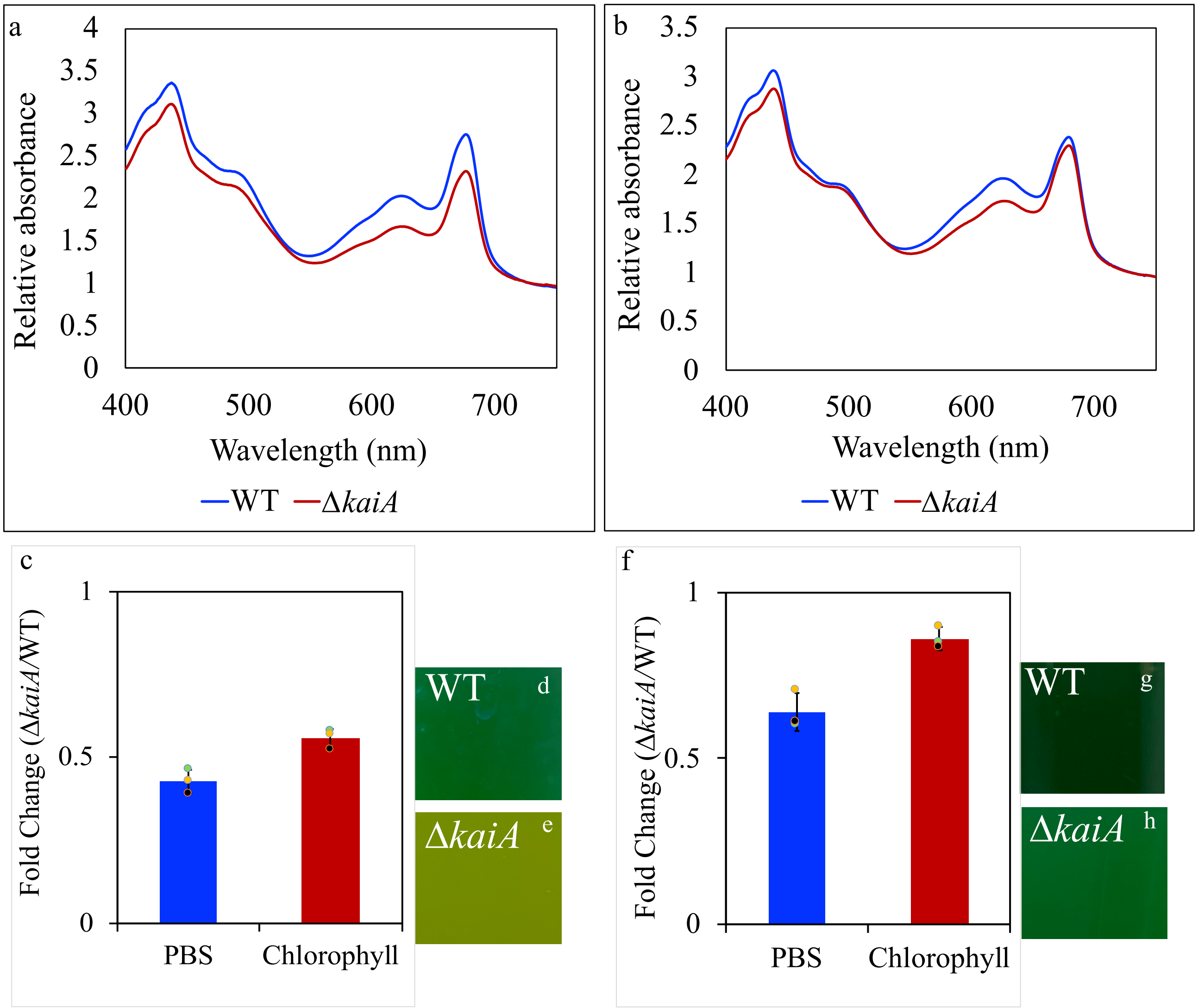

Fig. S8. Photosynthetic pigment analysis. Whole cell absorption spectra (a,b) and pigment estimation (c, f) for WT and Δ*kaiA* strains of *Cyanothece* 51142 grown under CL (a, c, d, e) and 12:12 LD (b, f, g, h) conditions in nitrogen deficient media. d,e,g,h are images showing the difference in pigment content between the WT and the mutant under nitrogen deficient growth conditions in multicultivators. A severely bleached mutant phenotype is observed under CL (d, e). Representative data are shown as the average of three biological replicates, and error bars show the standard deviation from the average.

Supplementary Figure 9

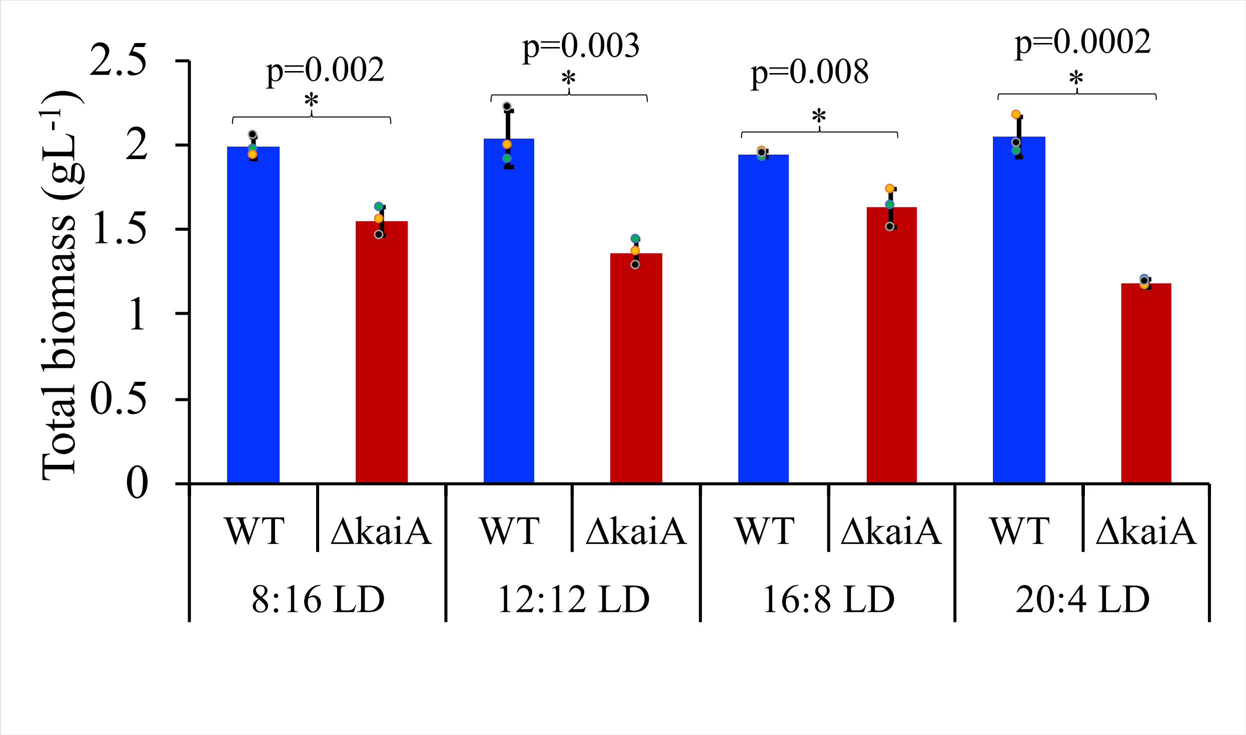

Fig. S9. Comparison of total biomass accumulation in the WT and the Δ*kaiA* mutant after cells grown under different LD regimes were subjected to CL. Samples were collected in triplicate at the end of the growth experiment for final biomass determination as described in the methods section. Error bars correspond to standard deviations from 3 biological replicates (*n*=3 samples).

Supplementary Figure 10

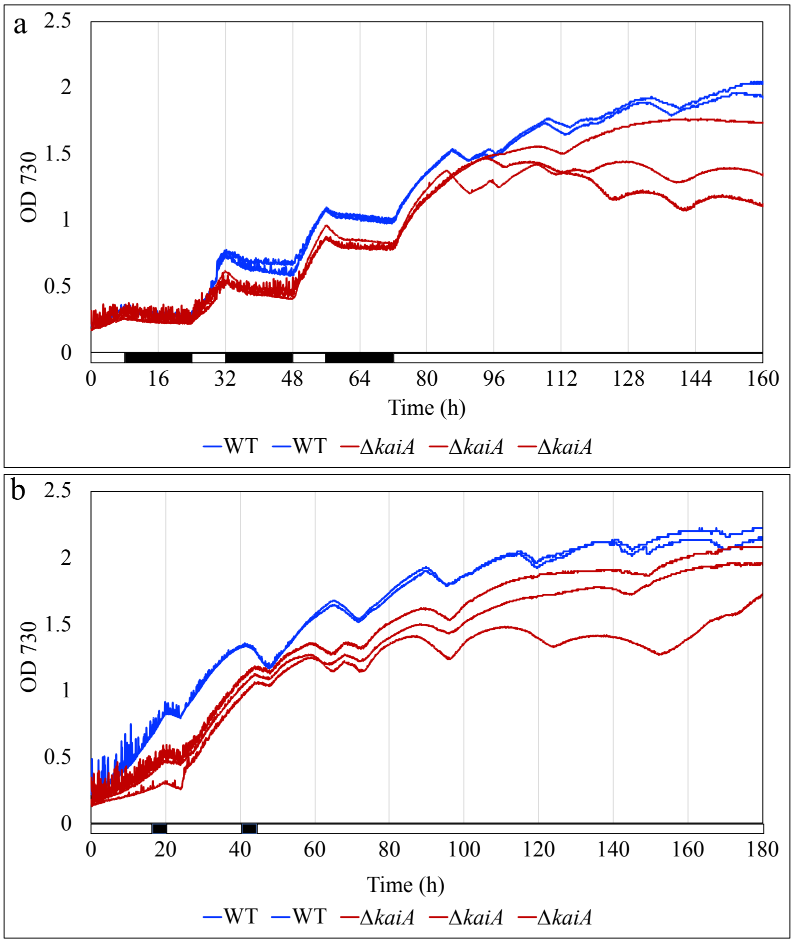

Fig. S10. Arrhythmic endogenous rhythms reflected in the growth phenotype of the Δ*kaiA* strain. Growth under 8:16 LD (a) and 20:4 LD (b) cycles followed by CL.

Supplementary Figure 11

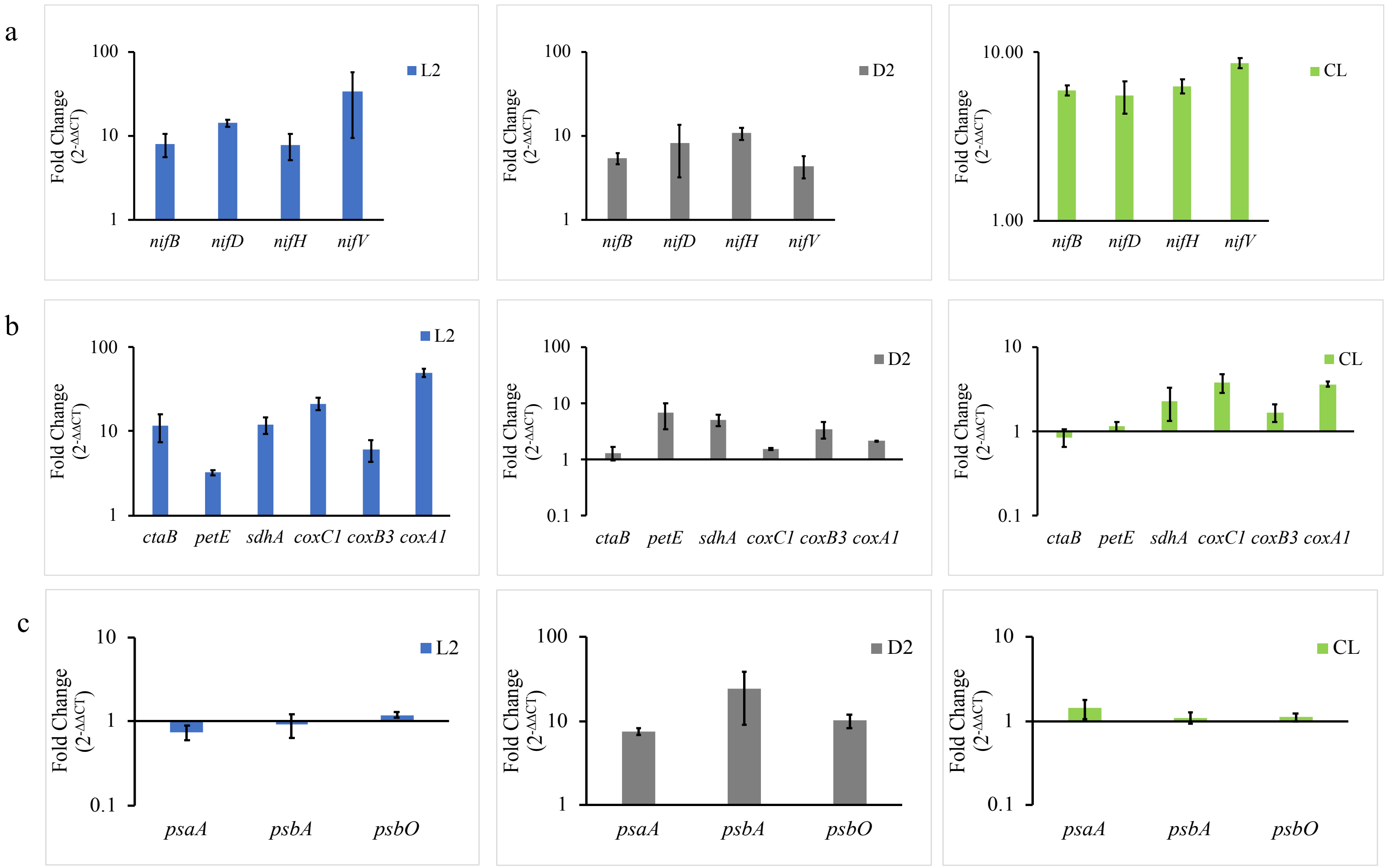

Fig. S11. Comparison of expression levels of representative genes from nitrogen fixation (a), respiration (b) and photosynthetic (c) pathways. Relative fold change ($2^{-\Delta\Delta Ct}$) in respective gene expression of Δ*kaiA* in relation to WT. Samples for RNA extraction were collected from both CL and LD (L2 and D2 time points) grown cultures. Error bars represent standard deviation of $2^{-\Delta\Delta Ct}$ values ($n = 3$) from the average.

Supplementary Figure 12

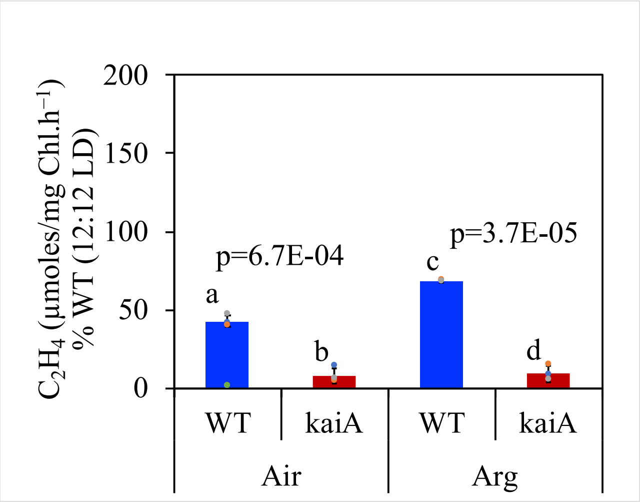

Fig. S12. Comparison of nitrogenase activity in the WT and Δ*kaiA* strains grown under 8:16 LD cycles. Activity is expressed as % WT activity under 12:12 LD growth. Representative data are shown as the average of three biological replicates (*n*=3 samples), and error bars show the standard deviation from the average.

Supplementary Figure 13

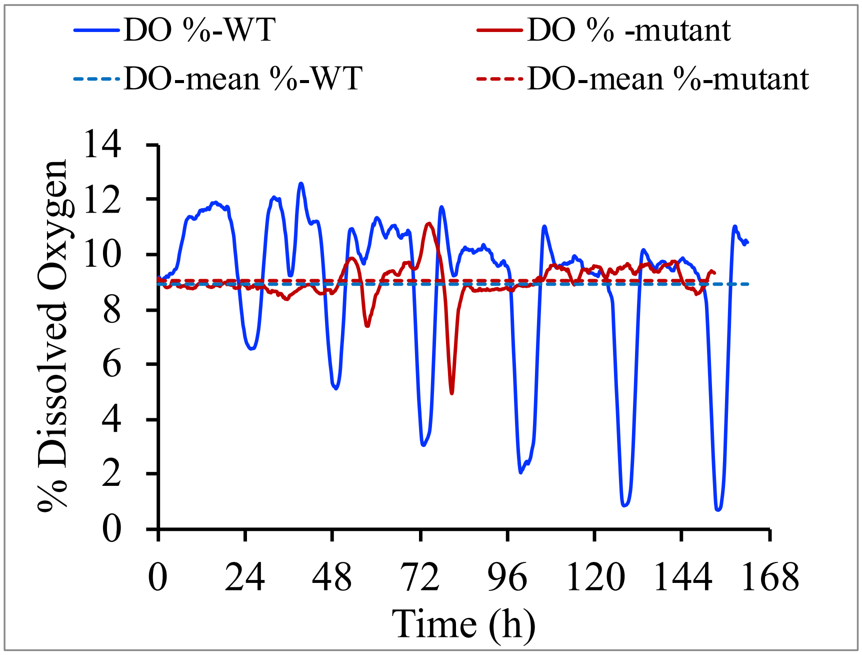

Fig. S13. Discerning oscillations in DO levels. To compare changes in DO levels, we computed the mean DO % levels as well as absolute change in the DO % over the entire CL growth period. One representative sample from the WT and Δ*kaiA* replicates with mean dissolved oxygen percent (DO%) of around 9% were selected for the quantitative analysis of the DO levels over the course of the experiment.

Supplementary Figure 14

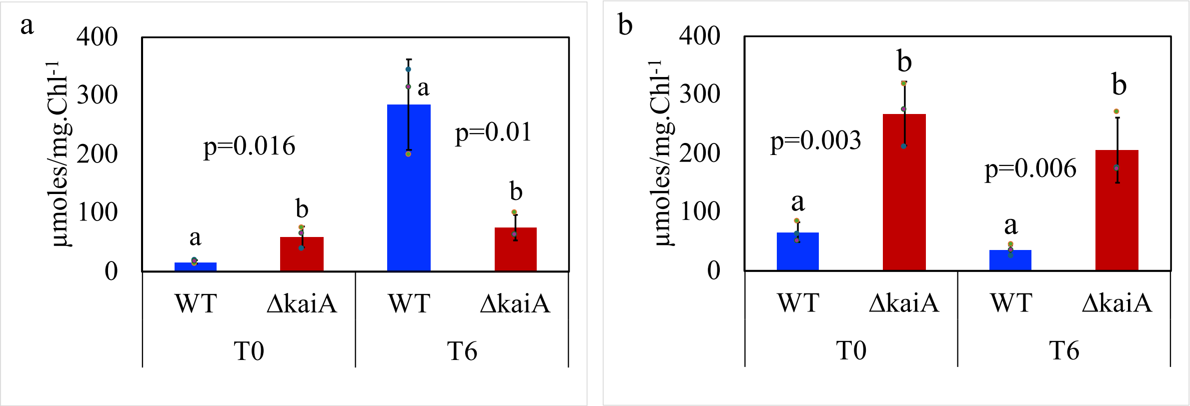

Fig. S14. Comparison of photosynthetic oxygen evolution (a) and respiratory oxygen uptake (b) in the WT and Δ*kaiA* strains. Oxygen evolution and oxygen uptake rates were measured in samples undergoing aerobic incubation for nitrogen fixation at the beginning of the incubation period (T0) and 6h after incubation in the subjective dark phase (T6). Representative data are shown as the average of three biological replicates (*n*=3 samples), and error bars show the standard deviation from the average. Asterisk * denote statistically different value of µ ($p < 0.05$)

Supplementary Figure 15

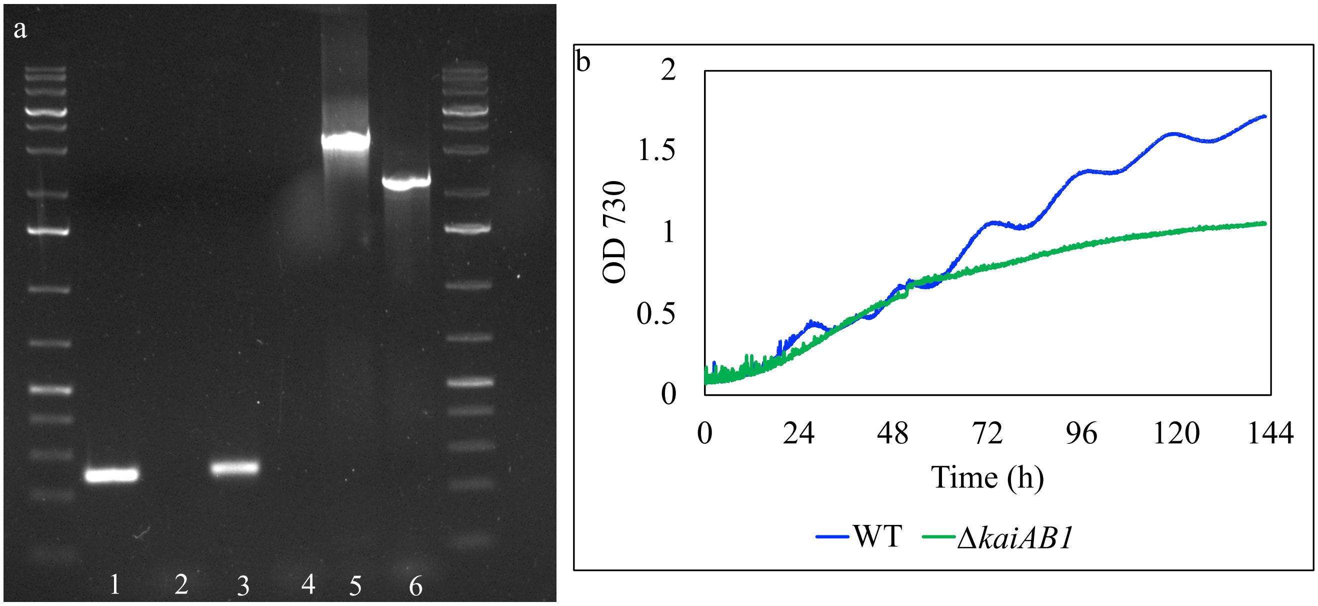

Fig. S15. Analysis of the Δ*kaiAB1* mutant of *Cyanothece* 51142 (a) PCR confirmation of the double deletion. 1- 4 – *kaiA* (1,2) and *kaiB1* (3,4) gene specific primers were used to amplify a 300 bp region within the WT (1), Δ*kaiA* (2), WT (3) and Δ*kaiB1* (4). 5 and 6 – Primers specific to regions upstream and downstream of the kaiAB1 genes were used. 4– WT, 5 - Δ*kaiAB1* (b) Growth comparison of WT and Δ*kaiAB1* double mutant. The mutant shows loss of rhythm observed in the growth pattern of the WT. Representative curves from 3 independent runs are presented.

Supplementary Figure 16

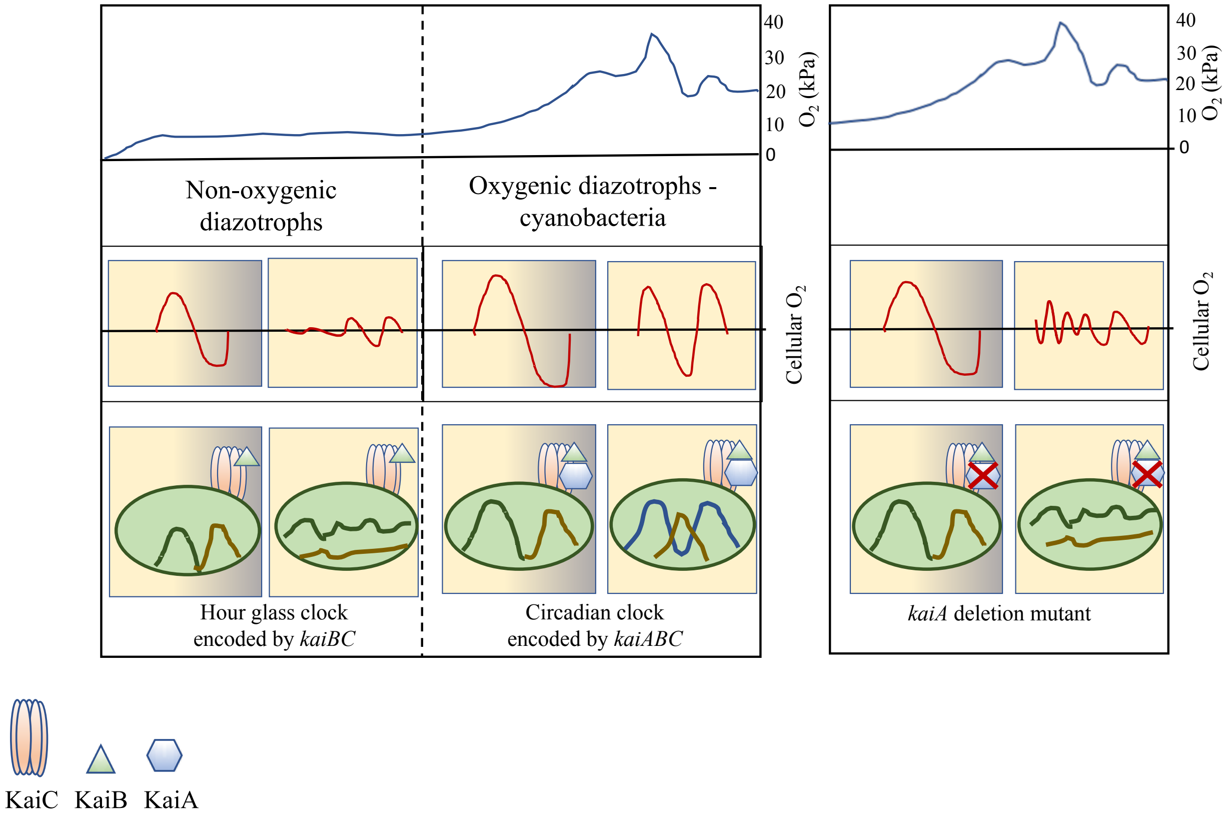

Fig. S16. Schematic representation of the importance of KaiA in regulating cellular oxygen dynamics in unicellular diazotrophic cyanobacteria. Under diurnal cycles, temporal separation of photosynthesis (green) and nitrogen fixation (brown) are enforced by external LD cues and KaiA is dispensable. Under CL the separation is controlled by the internal clock which enforces the rhythms of photosynthesis and nitrogen fixation and ensures optimal cellular oxygen levels (red) conducive for nitrogen fixation. KaiA is essential for the maintenance of robust self-sustained rhythms. With increasing oxygen levels in the atmosphere over the course of evolution, addition of KaiA to the KaiBC clock (which evolved from the KaiC oscillator based clock) was an adaptive strategy that unicellular diazotrophic cyanobacteria likely resorted to.

Supplementary Figure 17

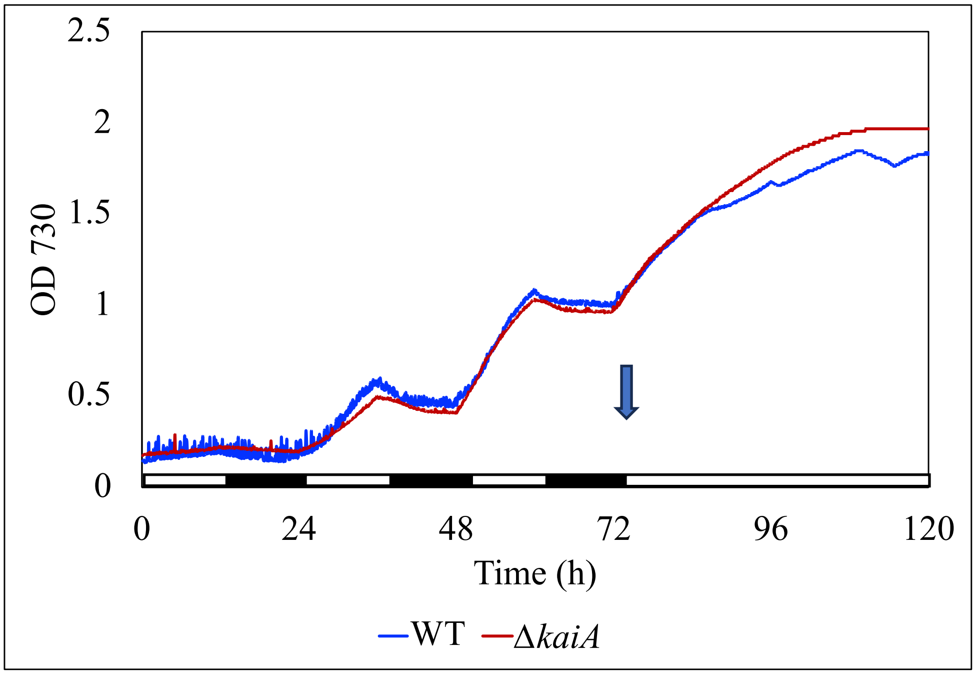

Fig. S17. Comparison of WT and Δ*kaiA* strains grown in nitrogen sufficient media under LD cycles followed by CL. Black bars represents dark phase of growth. Arrow points to point of transition to CL. Representative curves from 3 independent runs are presented.

Supplementary Figure 18

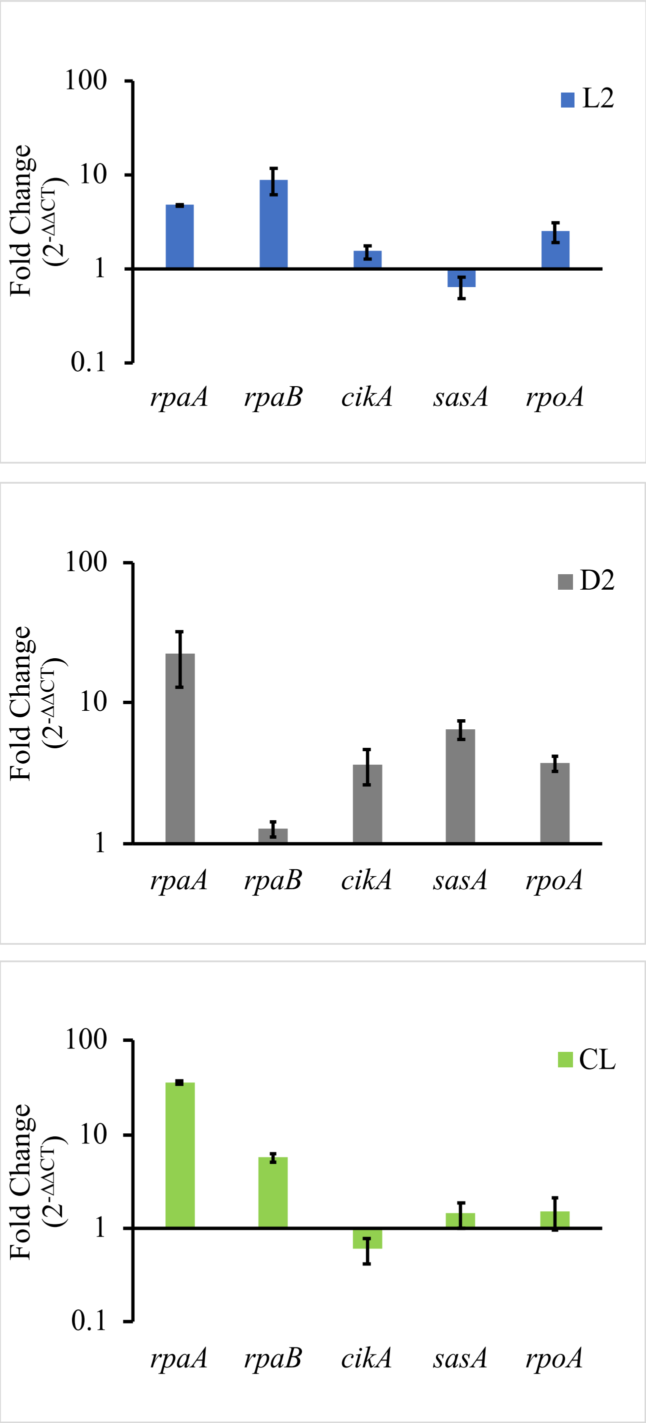

Fig. S18. Comparison of expression levels of certain regulatory genes of interest to circadian function based on studies in *Synechococcus* 7942. Relative fold change ($2^{-\Delta\Delta Ct}$) in expression of genes in the Δ*kaiA* mutant with respect to WT. Samples for RNA extraction were collected from both CL and LD (L2 and D2 time points) grown cultures. Error bars represent standard deviation of $2^{-\Delta\Delta Ct}$ values (n = 3) from the average.

Condition	Strain	Nitrogenase activity (μmoles C_2H_4 / mg Chl^{-1})	
		aerobic	anaerobic
CL	WT	8.2± 2.7	18.6±2.5
	Δ*kaiA*	0.7± .06	29.1±2.9
12:12 LD	WT	25.3±2.6	28.6±4.6
	Δ*kaiA*	20.6±2.6	34.7±4.1
16:8 LD	WT	26.4±4.1	30.5±1.4
	Δ*kaiA*	20.8±2.3	41.1±2.9
20:4 LD	WT	15.2±2.6	32.8±5.8
	Δ*kaiA*	6.8±0.9	45.2±2.6
8:16 LD	WT	11.4±1.3	21.2±0.4
	Δ*kaiA*	2.4±1.8	2.9±2.4

Table S1: Nitrogenase activity in the WT and Δ*kaiA* mutant grown in multicultivators under different light dark regimes and aerobic or anaerobic incubation conditions. ± values represent standard deviation from mean of 3 biological replicates (*n*=3 samples).

CRISPR-Cas12a approach	
kaiA-gRNA-F<br>*kaiA*-gRNA-R	5'-agatcaaacccgactctcaatttg<br>5'-agaccaaattgagagtcgggtttg
kaiA-up-repair-F<br>*kaiA*-up-repair-R	5'catttttttgtctagctttaatgcggtagttggtaccgctagaagtgttgcatcaaaatc<br>5'-ggccatctataaaggaggatctcaagaatgagagtacaactcct
kaiA-down-repair-F<br>*kaiA*-down-repair-R	5'-gaggagttgtactctcattcttgagatcctcctttatagatggcc<br>5'-cgctgccccggattacagatcctctagagtcgacggtaccggggattcctcaaaggtaac
Flanking primer -F<br>Flanking primer -R	5'-cgatccattaattgaaatagtcagc<br>5'-cgacgtagagtttgagaacatagg
gene specific – F<br>gene specific - R	5' ggtgcctttcttggaagatc<br>5'-gtaatggcacgatcaatggttac
kaiB1-gRNA-F<br>*kaiB1*-gRNA-R	5'-acttcaaaaaaacctatgtt<br>5'-aacataggtttttttgaagt
kaiB1-up-repair-F<br>*kaiB1*-up-repair-R	5'-gtctagctttaatgcggtagttggtacccgatgaacatctcacc<br>5'-ccttgaatcgaatcggactatttttctataaaggaggatcatctgaaaacatatc
kaiB1-down-repair-F<br>*kaiB1*-down-repair-R	5'-gatatgttttcagatgatcctcctttatagaaaaatagtccgattcgattcaagg<br>5'-cgctgccccggattacagatcctctagagtcgacggtaccgggaagatattaatgccatc
Flanking primer -F<br>Flanking primer -R	5'-ctatccacagaccattgcaaacc<br>5'-cattggatgatcgttgtgttaatct
gene specific – F<br>gene specific - R	5'-caaactctacgtcgctggtaatac<br>5'-cttcatagagaagatccaagcc
kaiC1-gRNA-F<br>*kaiC1*-gRNA-R	5'-agatgcccctaaaggtgttcgcaa<br>5'-agacttgcgaacacctttaggggc
kaiC1-up-repair-F<br>*kaiC1*-up-repair-R	5'-catttttttgtctagctttaatgcggtagttggtacccagaggtaaatcaagctattg<br>5'-caaaatttgtcaatgtctcaagactgctttataatcgttttataattaac
kaiC1-down-repair-F<br>*kaiC1*-down-repair-R	5'-gttaattataaaacgattataaagcagtcttgagacattgacaaattttg<br>5'-cgctgccccggattacagatcctctagagtcgacggtaccgtcatcacctgtaacacttcag
Flanking primer -F<br>Flanking primer -R	5'-ctccagaggataaacgagaattac<br>5'-cgagcattggatgatcgttgtg
gene specific – F<br>gene specific - R	5'-cgatgctgcatctgtggtgagac<br>5'-cgagcattggatgatcgttgtg
kaiAB1-gRNA-F<br>*kaiAB1*-gRNA-R	5'-agatcaaacccgactctcaatttg<br>5'-caaattgagagtcgggtttg
kaiAB1-up-repair-F<br>*kaiAB1*-up-repair-R	5'-gtctagctttaatgcggtagttggtacccttggttaagtatcgatccattaattg<br>5'-cgaaaccttgaatcgaatcggactatttttggtctgtggatagataaaaatcaag
kaiAB1-down-repair-F<br>*kaiAB1*-down-repair-R	5'-cttgatttttatctatccacagaccaaaaaatagtccgattcgattcaaggtttcg<br>5'-cgctgccccggattacagatcctctagagtcgacggtacccctttcatgtgggttgttccc
Flanking primer -F<br>Flanking primer -R	5'-cgatccattaattgaaatagtcagc<br>5'-cattggatgatcgttgtgttaatct
gene specific – F<br>gene specific - R	Gene specific primers for *kaiA* and *kaiB* (see above) were used
kaiAB1C1-gRNA-F (set1)<br>*kaiAB1C1*-gRNA-R	5'-agatcaaacccgactctcaatttg<br>5'-agaccaaattgagagtcgggtttg

kaiAB1C1-gRNA-F (set2)<br>*kaiAB1C1*-gRNA-R	5'-agatgcccctaaaggtgttcgcaa<br>5'-agacttgcgaacacctttaggggc
kaiAB1C1-up-repair-F<br>*kaiAB1C1*-up-repair-R	5'-cattttttgtctagctttaatgcggtagttggtaccgctagaagtgttgcatcaaaatc<br>5'-ggccatctataaaggaggatctcaagaatgagagtacaactcct
kaiAB1C1-down-repair-F<br>*kaiAB1C1*-down-repair-R	5'-gttaattataaaacgattataaagcagtcttgagacattgacaaattttg<br>5'-cgctgcccggattacagatcctctagagtcgacggtaccgtcatcacctgtaacacttcag
Flanking primer -F<br>Flanking primer -R	5'-cgatccattaattgaaatagtcagc<br>5'-cgagcattggatgatcgttgtg
gene specific – F<br>gene specific - R	Gene specific primers for kaiA, B1 and C1 (see above) were used
**Conventional approach – Kanamycin insertion**	
kaiC1-upstream-F<br>*kaiC1*-upstream-R	5'-ggcagaaattcgatatctagatctcgagcagaggtaaatcaagctattgatc<br>5'-ctggtatgagtcagcaacaccttctgactgctttataatcgttttataattaac
Kanamycin-F<br>Kanamycin-R	5'-Fgttaattataaaacgattataaagcagtcagaaggtgttgctgactcataccag<br>5'-cctttttacaaaatttgtcaatgtctcaattagaaaaactcatcgagcatcaaatg
kaiC1-downstream-F<br>*kaiC1*-downstream-R	5'-catttgatgctcgatgagtttttctaattgagacattgacaaattttgtaaaaaagg<br>5'-gtttgcgcaacgttgttgccattgctgcagcgatagagtcgacaccataaaggattttc
Flanking primer -F<br>Flanking primer -R	5'-ctccagaggataaacgagaattac<br>5'-cgagcattggatgatcgttgtg
gene specific – F<br>gene specific - R	5'-cgatgctgcatctgtggtgagac<br>5'-cgagcattggatgatcgttgtg

Table S2. Primers used to create clock mutants in *Cyanothece* 51142

nifB-F<br>*nifB*-R	5'-CAGTGAAGATGCCCATCATCAC<br>5'-GTTGAGTTCTTTAATGCGATCAACG
nifD-F<br>*nifD*-R	5'-GGATATGGTTCACATCTCCCACG<br>5'-GATGTGGTGTCCTAAGGACTGAG
nifH-F<br>*nifH*-R	5'-GAGAATCAACTATGCGTCAGATTGC<br>5'-CATCCAACTCCAGGCTCAGGAC
nifV-F<br>*nifV*-R	5'-GCGATCGCTTGTTTAATGGATG<br>5'-CGAATAAATCGTGATCTAGCGC
ctaB-F<br>*ctaB*-R	5'-GTGGACTCCTTTAGACTCTTTATTACC<br>5'GCATATAGAAGGCAATACCGGAC
petE-F<br>*petE*-R	5'CTCCAGCTTCTTCATTGCTGTTAG<br>5'-CTCCAGGCTCGTTGAAGGTTTC
sdhA-F<br>*sdhA*-R	5'-CACCCCACTGGGTTATATCCTG<br>5'-GTGGCGCAAGTCGAGATAGAC
coxC1-F<br>*coxC1*-R	5'-CCCTGTTTTTAGTGGCGGAAAG<br>5'-CTGGCGAATAAATTGGTCGTTAATC
coxB3-F<br>*coxB3*-R	5'-GAAGGGAATGTCACCTTAGAAGTGG<br>5'-CACATCTTCTGAGGTTAACGCTAAC
coxA1-F<br>*coxA1*-R	5'-CCAATTTATGACCCTACATGGCAC<br>5'-CCAACAAAACAGAGGCATACTGTG
psaA-F<br>*psaA*-R	5'-CTTTAGCGCGCACTTCGGTC<br>5'-GAACCAGCCAGCAAACAGCATTAG
psbA-F<br>*psbA*-R	5'-GGTTTGGAGTCTTAATGATCCCAACAC<br>5'-CCAAGGCCGCATTCCTAATCGATAAC
psbO-F<br>*psbO*-R	5'-TGGATGATCTGTGTCTCGAACC<br>5'-GTTCTCCTCCAGGTAACTGAACG

Table S3. Primers used for Q-RTPCR analysis
